# Body Mass Index Differences in the Gut Microbiota Are Gender Specific

**DOI:** 10.3389/fmicb.2018.01250

**Published:** 2018-06-22

**Authors:** Xuefeng Gao, Meirong Zhang, Junmian Xue, Jiandong Huang, Rihong Zhuang, Xiaolin Zhou, Huayue Zhang, Qiang Fu, Yi Hao

**Affiliations:** ^1^Shenzhen HRK Bio-tech Co., Ltd., Shenzhen, China; ^2^Shenzhen University General Hospital, Shenzhen, China; ^3^Department of Pathogen Biology, School of Basic Medicine, Tongji Medical College, Huazhong University of Science and Technology, Wuhan, China; ^4^Tongji Hospital, Tongji Medical College, Huazhong University of Science and Technology, Wuhan, China

**Keywords:** obesity, gut microbiota, 16S rRNA, Chinese, gender

## Abstract

**Background:** The gut microbiota is increasingly recognized as playing an important role in the development of obesity, but the influence of gender remains elusive. Using a large cohort of Chinese adults, our study aimed to identify differences in gut microbiota as a function of body mass index (BMI) and investigate gender specific features within these differences.

**Methods:** Five hundred fifty-one participants were categorized as underweight, normal, overweight, or obese, based on their BMI. Fecal microbiome composition was profiled via 16S rRNA gene sequencing. Generalized linear model (GLM), BugBase, PICRUSt, and SPIEC-EASI were employed to assess the variabilities in richness, diversity, structure, organism-level microbiome phenotypes, molecular functions, and ecological networks of the bacterial community that associated with BMI and sex.

**Results:** The bacterial community of the underweight group exhibited significantly higher alpha diversity than other BMI groups. When stratified by gender, the pattern of alpha diversity across BMI was maintained in females, but no significant difference in alpha diversity was detected among the BMI groups of males. An enrichment of Fusobacteria was observed in the fecal microbiota of obese males, while obese females demonstrated an increased relative abundance of Actinobacteria. Analysis of microbial community-level phenotypes revealed that underweight males tend to have more anaerobic and less facultatively anaerobic bacteria, indicating a reduced resistance to oxidative stress. Functionally, butyrate-acetoacetate CoA-transferase was enriched in obese individuals, which might favor energy accumulation. PhoH-like ATPase was found to be increased in male obese subjects, indicating a propensity to harvest energy. The microbial ecological network of the obese group contained more antagonistic microbial interactions as well as high-degree nodes.

**Conclusion:** Using a large Chinese cohort, we demonstrated BMI-associated differences in gut microbiota composition, functions, and ecological networks, which were influenced by gender. Results in this area have shown variability across several independent studies, suggesting that further investigation is needed to understand the role of the microbiota in modulating host energy harvest and storage, and the impact of sex on these functions.

## Introduction

The prevalence of obesity worldwide has reached 5% in children and 12% in adults, more than doubling since 1980 (Gregg and Shaw, 2017). Even more alarming is the observation that obesity rates have almost tripled in youth and young adults from middle-income countries such as China. Obesity has been shown to increase morbidity and mortality when associated with many diseases, including type 2 diabetes, cardiovascular disease, pulmonary hypertension, and cancer (Pi-Sunyer, 2009). The development of obesity appears to be related to a complex set of interactions between genetics, host physiology, and environmental factors. More recently, the microbiota of the gastrointestinal tract is being increasingly recognized as an important factor in the development of obesity (Ley et al., 2006; Shen et al., 2013; John and Mullin, 2016). The gut microbiota plays an important role in energy harvest, nutrient extraction, resistance to infection, and immunomodulation, thereby, influencing human physiology (Rios-Covian et al., 2016). Although the exact mechanisms by which the gut microbiota contributes to obesity are unclear, it is well established that modification of the gut microbiota can increase energy production, trigger low-grade inflammation, induce insulin resistance, and affect fatty acid tissue composition (Musso et al., 2010).

Comparisons between gut microbiota profiles of obese and normal/underweight individuals reveal distinctions in the composition of bacterial communities. For example, Ley et al. (2006) and Turnbaugh et al. (2009) found a lower relative abundance of Bacteroidetes and reduced biodiversity in the gut microbiota of obese individuals. Conflicting results, however, have been reported from several other studies. These findings include no difference between lean and obese individuals with respect to the relative abundance of Actinobacteria, Bacteroidetes, or Firmicutes (Duncan et al., 2008; Jumpertz et al., 2011), a higher relative abundance of Bacteroidetes in obese (Patil et al., 2012) or overweight (Schwiertz et al., 2010) individuals compared with lean controls, and increased Firmicutes and Actinobacteria coupled with decreased Proteobacteria and Fusobacteria in obese compared to normal-weight individuals (Piombino et al., 2014). Some studies have described variability in the species that are associated with obesity. *Bifidobacterium* strains appear to be reduced in overweight, obese, or type 2 diabetic patients compared to lean subjects (Schwiertz et al., 2010; Wu et al., 2010; Million et al., 2012); in the fecal samples of normal-weight children who became overweight, a higher level of *Staphylococcus aureus* and a lower level of *Bifidobacterium* were observed (Kalliomaki et al., 2008); abundances of *Faecalibacterium prausnitzii* and *Flavonifractor plautii* negatively correlated with body mass index (BMI) (Borgo et al., 2018). More recently, *Bacteroides thetaiotaomicron*, a glutamate-fermenting commensal, was found to be markedly reduced in obese Chinese individuals (Liu et al., 2017).

The composition of the gut microbiome appears to be determined by both genetics (Goodrich et al., 2014) and environmental factors, including gender, age, geographic location, and diet (Li et al., 2008; Escobar et al., 2014; Andoh et al., 2016). Escobar et al. (2014) showed that the gut microbiota of Colombians differs from that of Asians, Americans, and Europeans; Andoh et al. (2016) observed that obesity-associated gut microbiota in Japanese individuals is different from that found in Western subjects; Li et al. (2008) showed that among Chinese individuals, Clostridia, Bacteroidetes, and Proteobacteria are more abundant in males than females. Due to inconsistent findings and small sample sizes used in these studies, further investigations with larger populations are wanted. In this study we analyzed of the gut microbiota profile of 551 Chinese adults, aiming to provide further evidence of the characteristics of gut microbiota associated with BMI and gender.

## Materials and Methods

### Study Cohort

Sample collection was approved by the Ethics Committee of Tongji Medical College, Huazhong University of Science and Technology, and all participants provided written informed consent. The study was conducted in compliance with the Declaration of Helsinki. Study exclusion criteria were as follows: (1) diabetes; (2) chronic diarrhea or constipation; (3) long-term use of medication (e.g., antihypertensive drugs); (4) antibiotic use in the 2 months prior to sampling. After DNA sequences were obtained, samples with less than 10,000 reads were filtered out. Finally, samples from 551 subjects were used in this study. General characteristics of the subjects are shown in **Table 1**, including sex, age, and BMI (kg/m^2^) of each group. Volunteers were classified into four BMI categories according to the WHO Asian BMI cut points (WHO Expert Consultation, 2004) (underweight: <18.5 kg/m^2^; normal: 18.5–23 kg/m^2^; overweight: 23–27.5 kg/m^2^; obese: ≥27.5 kg/m^2^).

**Table 1:** Characteristics of the study population categorized by BMI.

	Underweight		Normal		Overweight		Obese	
	Female	Male	Female	Male	Female	Male	Female	Male
Subjects	49	13	168	93	55	115	20	38
Age (years)	38.0 ± 25.6	21.5 ± 5.5	35.6 ± 14.3	37.8 ± 17.3	38.1 ± 12.6	41.7 ± 15.9	35.5 ± 12.7	34.7 ± 12.5
BMI (kg/m^2^)	17.5 ± 1.0	16.7 ± 1.1	20.7 ± 1.3	21.3 ± 1.3	24.7 ± 1.3	25.1 ± 1.2	31.7 ± 4.3	31.2 ± 3.2


### Sample Collection, DNA Extraction, and 16S rRNA Gene Sequencing

Fecal samples were self-collected by the volunteers using a 1.5 mL vial containing 1.0 mL inhibit EX Buffer (Qiagen, Germany). Samples were shipped to HRK-biotech lab for DNA extraction and sequencing within 72 h of collection.

Microbiome community profiling and sequencing were performed as described (Wang et al., 2018). In brief, genomic DNA was extracted with QIAamp Fast DNA Stool Mini Kit (Qiagen, Germany), following recommendations of the International Human Microbiome Standards^1^. DNA was quantified using a dsDNA HS assay on a Qubit 3.0 (Thermo Fisher Scientific, United States). The universal primer set 341F/806R was used to amplify the isolated genomic DNA for the V3–V4 hypervariable regions of 16S rRNA genes. The Pooled amplicon libraries were sequenced on the MiSeq platform (Illumina, Inc., San Diego, CA, United States) using the 2×300 bp paired-end protocol yielding paired-end reads with approximately 50 bp overlapping sequence.

### 16S rRNA Gene Sequencing Analysis

Sequencing data was processed using the QIIME 1.9.1^2^ data curation pipeline. We removed human contaminated sequences with KneadData^3^. Chimeric sequences were identified and removed using UCHIME (Edgar et al., 2011). Operational taxonomic units (OTUs) were clustered using a closed-reference picking protocol with the UCLUST algorithm based on 97% nucleotide similarity. Microbial OTUs were annotated with the Greengenes database release 13_8^4^.

The statistical analysis of bacterial communities was performed with SHAMAN (Quereda et al., 2016). OTU counts were normalized by taking into account the confounding effect of sex and age, and adjusting for age when investigating gender-specific differences in gut microbiota. The richness of the microbial community is estimated by the number of observed OTUs. The Shannon diversity index, the Simpson index, and the Inverse Simpson index were used as measures of alpha diversity. Principal Coordinate Analysis (PCoA) of samples by weighted and unweighted UniFrac distance were used to evaluate overall differences in beta-diversity between the microbiomes. The data were analyzed using a generalized linear model (GLM) and contrast vectors were defined to assess the significance of differences between sample groups. Resulting *p*-values were adjusted for multiple testing according to the Benjamini–Hochberg procedure.

The relative representation of microbiome characteristics were predicted using BugBase (Ward et al. unpublished) on the basis of six phenotype categories: Gram staining, oxygen tolerance, ability to form biofilms, mobile element content, pathogenicity, and oxidative stress tolerance. This tool leverages Integrated Microbial Genomes (IMG4) (Markowitz et al., 2012), the Kyoto Encyclopedia of Genes and Genomes (KEGG) database (Kanehisa et al., 2012), and the Pathosystems Resource Integration Center (PATRIC) (Snyder et al., 2007), to identify the contribution of specific OTUs to a community-level phenotype.

Phylogenetic Investigation of Communities by Reconstruction of Unobserved States (PICRUSt) (Langille et al., 2013) was used to analyze metagenomes which make functional predictions based on the Greengenes 16S rRNA database and KEGG orthologs. Functional differences among the four BMI groups were compared using STAMP software^5^ (Parks et al., 2014). ANOVA with the Tukey–Kramer test and the Benjamini–Hochberg correction were used for multiple-group analysis.

Sparse InversE Covariance estimation for Ecological Association (SPIEC-EASI) (Kurtz et al., 2015) was applied to infer microbiota association networks from the numerous OTUs, with *lambda.min.rati*o = 0.01, *nlambda* = 20.

## Results

### Characteristics of Gut Microbiota in Obese Chinese

**FIGURE 1:**
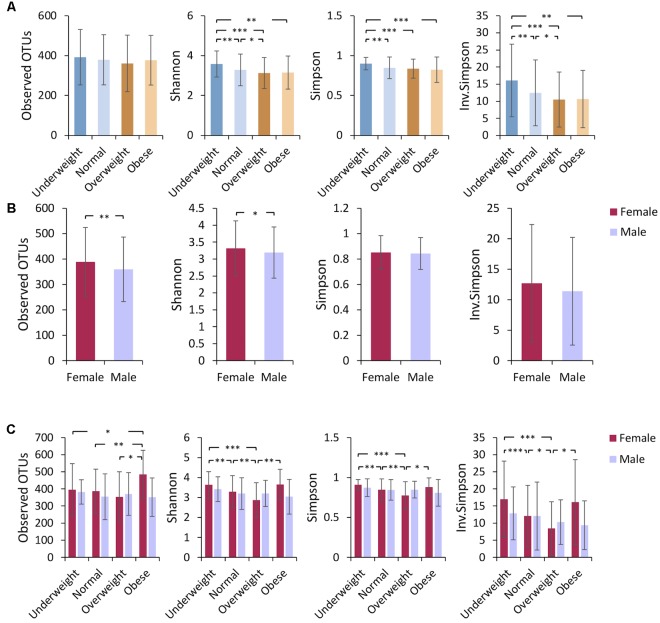
Underweight individuals tend to have higher microbiome alpha diversity. Alpha diversity (mean ± standard deviation) was compared **(A)** among different BMI groups, **(B)** between females and males, and **(C)** among gender-specific BMI groups. Bacterial community richness was defined by the observed number of OTUs and alpha diversity was calculated using the Shannon index, Simpson index, and Inverse Simpson index. Paired *t*-tests were performed for alpha diversity measures. The *P*-value ranges are: ^∗^*P* < 0.05, ^∗∗^*P* < 0.01, and ^∗∗∗^*P* < 0.001.

Comparative analysis revealed that the richness (measured by the number of observed OTUs) of gut bacterial communities was not significantly different among the four BMI groups (**Figure 1A**). A higher alpha diversity (measured by the Shannon, Simpson, and Inverse-Simpson indexes) was observed in underweight individuals as compared to other groups with higher BMIs (**Figure 1A**), a finding consistent with previous studies (Turnbaugh et al., 2009; Remely et al., 2014; Andoh et al., 2016). No significant difference in alpha diversity was observed between obese and normal-weight sample groups (**Figure 1A**). The analysis of beta diversity revealed that fecal microbial communities of the four BMI groups were not distinct from each other (Supplementary Figure S1A), indicating low among-group dissimilarities.

**FIGURE 2:**
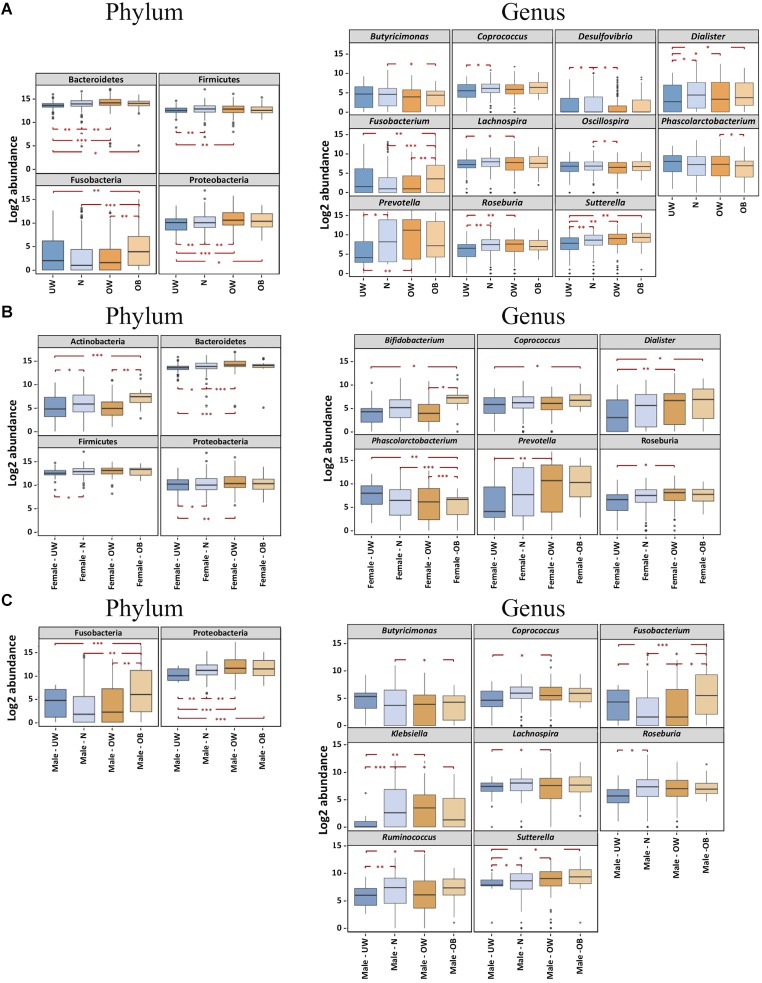
BMI differences in the gut microbiota at the phylum and genus levels were influenced by gender. Bacterial phyla and genera demonstrated significantly different log_2_ abundances across the four BMI groups in **(A)** the overall cohort, **(B)** females and **(C)** males. Lower and upper limits of the boxes represent 25th and 75th percentiles, respectively. Whiskers represent 1.5^∗^ inter-quartile range. *P*-value ranges are: ^∗^*P* < 0.05, ^∗∗^*P* < 0.01, and ^∗∗∗^*P* < 0.001. UW, underweight; N, normal; OW, overweight; OB, obese.

Inter-group comparisons of taxonomic profiles revealed that higher proportions of Bacteroidetes, Fusobacteria, and Proteobacteria were observed in obese individuals compared to those who were underweight (**Figure 2A**). In addition, these groups demonstrated no significant differences in the relative abundance of Actinobacteria (data not shown) or Firmicutes. At the genus level, *Fusobacterium* was more abundant in the obese group (**Figure 2A**). Underweight subjects had lower abundances of *Dialister* and *Sutterella* than other BMI groups (**Figure 2A**). BugBase (Ward et al. unpublished) was used to infer and compare organism-level microbiome phenotypes among the four BMI groups. We observed that a significantly higher representation of aerobic bacteria in the obese group (Supplementary Figure S3). Additionally, the normal-weight group was shown to have more Gram-positive bacteria (Supplementary Figure S3).

**FIGURE 3:**
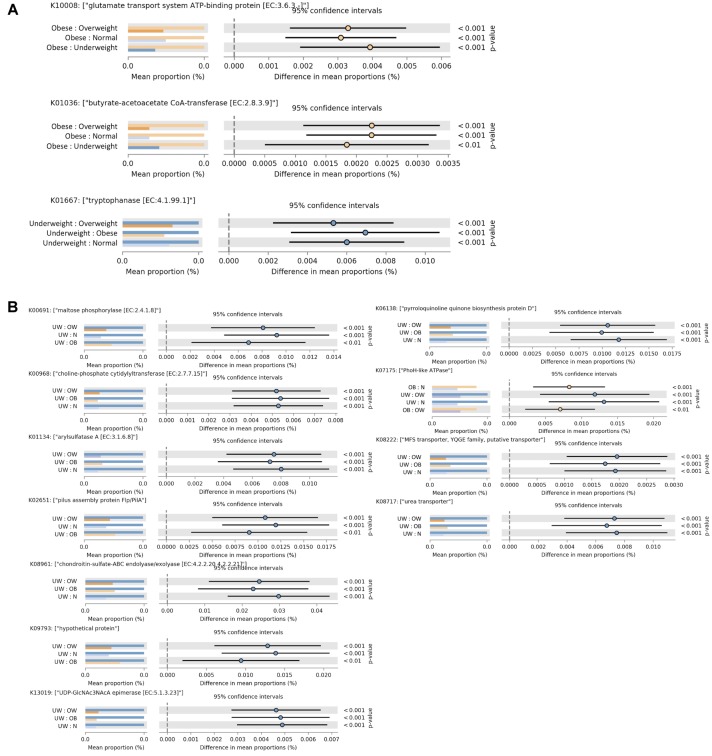
Functional divergence of gut microbiota across different BMI groups. Significant differences in functional orthologs were identified among the four BMI groups in **(A)** the overall cohort and **(B)** males. ANOVA was applied with a *post hoc* test (Tukey–Kramer at 0.95), and a multiple-test correction of Benjamini–Hochberg FDR (corrected *p*-value (<0.005). Each bar plot indicates the mean proportion of sequences assigned to a feature in each group. Extended error bars indicate the difference in mean proportion between the two groups along with the associated confidence interval of this effect size and the *p*-value of the specified statistical test. UW, underweight; N, normal; OW, overweight; OB, obese.

To evaluate functional differences in the gut microbiota among the BMI groups, functional profiles of the fecal microbiome were predicted using PICRUSt (Langille et al., 2013). Tryptophanase, which is involved in metabolizing tryptophan into indole, was found to be enriched in the underweight group (**Figure 3A**). In the obese group, two functional profiles were significantly elevated (**Figure 3A**). Butyrate-acetoacetate CoA-transferase, involved in butyrate metabolism, was found at higher levels in obese adults. Increased glutamate transport system ATP-binding protein (**Figure 3A**) was also observed in obese individuals. This protein may be involved in regulating host glutamate levels, and excessive glutamate consumption has been positively correlated with overweight in Chinese adults (He et al., 2011).

### BMI Differences in the Gut Microbiota Are Gender Specific

Gender-based differences in host gut microbiota composition have been reported in several studies (Markle et al., 2013; Haro et al., 2016). In our investigation, the overall gut microbiota community composition (measured by beta diversity) was not significantly different between males and females (Supplementary Figure S1B), while a higher alpha diversity was found in the fecal microbiota of female subjects (**Figure 1B**). We investigated whether the relative abundance of specific taxa might differ between Chinese men and women. Regardless of BMI, we did not find significant taxonomic differences at the level of phylum, class, order, or family between men and women. However, at the genus level, *Ruminococcus* was significantly more abundant in fecal samples from women compared to men (Supplementary Figure S2).

We then asked if BMI differences in gut microbiota composition varies between females and males. To examine this, we performed inter-group comparisons between different BMI categories, separating males and females. PCoA (on OTU-level abundance) did not distinguish the gender-specific BMI groups (Supplementary Figure S1C). Among Chinese females, alpha diversity was similar in fecal microbiota of underweight and obese individuals but was lower in overweight subjects (**Figure 1C**). Obese female subjects had higher relative amounts of the genera *Bifidobacterium* (belonging to the Actinobacteria phylum), *Coprococcus*, and *Dialister*, while exhibiting a lower relative abundance of *Phascolarctobacterium* (**Figure 2B**). Both underweight females and males demonstrated reduced proportions of Proteobacteria (**Figures 2B,C**). For Chinese males, there was no significant difference in alpha diversity among subjects with different BMIs (**Figure 1C**). *Fusobacterium* (belonging to the Fusobacteria phylum) was enriched in obese than underweight male subjects, and *Sutterella* was depleted in underweight males (**Figure 2C**).

In terms of microbiome phenotypes, underweight males were found to have a greater abundance of anaerobic bacteria, and lower amounts of facultatively anaerobic bacteria, indicating the community was relatively less resistant to oxidative stress (Supplementary Figure S3). In addition, Gram-positive bacteria were observed to be significantly enriched in normal-weight females (Supplementary Figure S3).

PICRUSt analysis of bacterial communities revealed that several KEGG pathways associated with metabolic functions were enriched in the underweight male subjects (**Figure 3B**). In particular, an ATPase complex was highly represented in both obese and underweight male individuals. In female subjects there was no statistically significant difference in gut microbial functions among the four BMI groups.

### Inference of Microbial Ecological Networks With Different BMI

**FIGURE 4:**
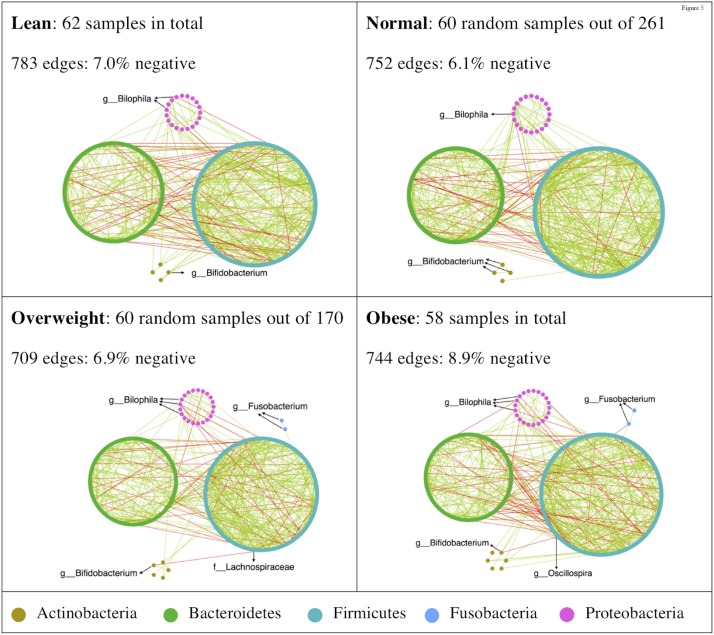
High-degree nodes and co-exclusion relationships were enriched in microbial ecological network of the obese group. SPIEC-EASI was used for networks construction. Network visualizations with OTU nodes were colored according to phylum lineage and grouped in circles. Edges are colored by sign (co-occurrence: green; co-exclusion: red).

To determine whether there were variations in the organization of microbial communities in different BMI groups, we investigated bacterial interactions with SPIEC-EASI (Kurtz et al., 2015). The inferred networks were dominated by co-occurrence relationships (**Figure 4**), while the obese group tending to harbor more co-exclusion relationships in the bacterial community. Antagonistic relationships between *Bilophila* and other taxa were consistently observed in all BMI groups (**Figure 4**). *Bifidobacterium* was negatively correlated with Lachnospiraceae and *Oscillospira* in overweight and obese groups, respectively (**Figure 4**). Additionally, *Fusobacteria* only appeared in the networks of overweight and obese groups (**Figure 4**).

The distribution of the network node degrees was similar among different BMI groups (Supplementary Figure S4). Some hubs (a node with higher than10 edges) were observed in the normal and obese BMI groups (Supplementary Figure S4). We estimated network stability by removing nodes from the static network and assessing how quickly the robustness degraded. The natural connectivity was highest in the normal-weight group, which decreased more rapidly than other groups (Supplementary Figure S5). However, with approximately 75% of nodes removed, the network from the normal-weight group exhibited better robustness than its BMI counterparts (Supplementary Figure S5).

## Discussion

The gut microbiota is increasingly identified as making a crucial contribution to energy homeostasis and low-grade inflammation, thus likely participating the development of obesity and metabolic disease (Backhed et al., 2004; Turnbaugh et al., 2006, 2008). This study aimed to identify differences in the gut microbiota associated with BMI and gender. We demonstrated that obese individuals have less diverse gut microbiota than those underweight, in accordance with previous studies (Turnbaugh et al., 2009; Remely et al., 2014; Andoh et al., 2016). We did not observe, however, lower gene/OTU counts in the obese group as demonstrated in another study with a younger Chinese cohort (median age = 23.2 ± 1.8 years) (Liu et al., 2017). Numerous studies have described an increase of Firmicutes and a decrease of Bacteroidetes (i.e., a high ratio of Firmicutes to Bacteroidetes) in the obese subjects. However, inconsistent results have been reported. For example, some studies found no difference in the relative abundances of these two phyla upon comparison of obese and lean subjects (Duncan et al., 2008; Jumpertz et al., 2011). Using a large Chinese cohort, we found that Bacteroidetes was enriched in obese subjects compared to underweight subjects, which is in agreement with some previous studies (Schwiertz et al., 2010; Patil et al., 2012). Fusobacteria was found to be significantly enriched in the obese group, which was also confirmed at the genus level. This finding was in line with a previous study with Japanese subjects (Andoh et al., 2016), but was not observed in another study with a large Korean cohort (*n* = 1463) (Yun et al., 2017), suggesting that it may not be related to genetic similarity. A meta-analysis of 10 individual studies did not find an association between BMI and microbiome composition (Sze and Schloss, 2016). Taken together, these disparites indicate that the composition of the gut microbiota is not sufficient to characterize populations with variant BMIs.

We used BugBase to infer microbial community phenotypes, including Gram staining, oxygen tolerance, biofilm formation, mobile element content, pathogenicity, and oxidative stress tolerance. We observed that the gut microbiota of the obese group supported a higher abundance of aerobic bacteria. Assessment of predicted metabolic functions revealed that three functional orthologs were increased in obese subjects. Included among these was butyrate-acetoacetate CoA-transferase, an enzyme involved in host energy balance through butyrate metabolism and glutamate transport. In the context of obesity, short-chain fatty acids (SCFAs) such as butyrate have been shown to exhibit both obesogenic and anti-obesity effects (Samuel et al., 2008; den Besten et al., 2013). Enrichment of butyrate-acetoacetate CoA-transferase in obese subjects suggests that butyrate may be implicated in energy accumulation.

Network analysis showed that the microbial ecological network of the obese group contained relatively more high-degree hubs and an increased proportion of antagonistic bacterial relationships. There was a positive correlation between *Bifidobacterium* and other bacteria in under- and normal-weight groups. In overweight and obese Chinese, antagonistic relationships were observed between *Bifidobacterium* and both Lachnospiraceae and *Oscillospira*. Some Lachnospiraceae bacteria have been found to contribute to metabolic disorders like type 2 diabetes (Kameyama and Itoh, 2014). Thus, the co-exclusion relationship between *Bifidobacterium* and potential pathogenic bacteria such as Lachnospiraceae may reduce the risk of metabolic disorders.

Sex-related differences in gut microbiota have previously been reported (Li et al., 2008; Markle et al., 2013; Haro et al., 2016; Borgo et al., 2018). The genera *Bilophila*, *Veillonella*, and *Methanobrevibacter* were previously found to have distinct abundances in European men and women (Haro et al., 2016). In our study, the representation of these genera did not differ significantly between males and females. Although the overall gut microbiota composition of men and women was indistinguishable, we found that there was a greater abundance of *Ruminococcus* in female subjects. Interestingly, this genus was also found to be more abundant in female mice (Org et al., 2016). We then considered the impact of sex on BMI differences in the gut microbiota composition. Inter-group comparisons revealed that *Fusobacterium* was enriched in obese Chinese males, whereas obese Chinese females were characterized by increased abundances of the *Bifidobacterium*, *Coprococcus*, and *Dialister* genera and a decreased number of *Phascolarctobacterium*. Notably, lower levels of *Bifidobacterium* have been reported in obese subjects in multiple studies (Schwiertz et al., 2010; Wu et al., 2010; Million et al., 2012). This may suggest an association between obesity and *Bifidobacterium* at the species level. With regard to bacterial community phenotypes, we found that underweight males tended to have more anaerobic and less facultatively anaerobic bacteria, leading to a reduced resistance to oxidative stress. Underweight males differ significantly from other BMI groups of males with regard to multiple KEGG functional orthologs. In particular, PhoH-like ATPase was enriched in both underweight and obese males. Interestingly, the abundance of this enzyme in both of these populations may indicate that pathways related to energy harvest do not sufficiently explain host obesity (Musso et al., 2010).

## Conclusion

Although the relationship between gut microbiota and obesity are increasingly reported, studies involving large-scale cohorts remain rare. Using 16S rRNA gene sequencing data from 551 Chinese adults, we demonstrated statistically significant differences in gut bacterial community diversity, composition, phenotypes, functions, and ecological networks. These diverse profiles were associated with BMI but were also sex-specific. Both parallels and distinctions were observed between our study and others in terms of obesity-associated gut microbiota composition. These data support an important role of the microbiota as it functions to modulate host energy harvest and storage, and gender differences should be taken into account.

## Author Contributions

YH and XG designed the study. JX, JH, RZ, XZ, MZ, and HZ carried out experiments. XG performed the data analysis. XG, MZ, and YH wrote the manuscript. QF read and revised the manuscript.

## Conflict of Interest Statement

The authors declare that the research was conducted in the absence of any commercial or financial relationships that could be construed as a potential conflict of interest.

## Footnotes

1 http://www.microbiome-standards.org

2 http://qiime.org/

3 https://bitbucket.org/biobakery/kneaddata/wiki/Home

4 http://greengenes.secondgenome.com

5 http://kiwi.cs.dal.ca/Software/STAMP
