# Supplementary material for: Body Mass Index Differences in the Gut Microbiota Are Gender Specific
Source: Front Microbiol. 2018 Jun 22;9:1250. doi: 10.3389/fmicb.2018.01250 (PMC6023965; doi:10.3389/fmicb.2018.01250)
Supplement: Supplementary file 1 [file Data_Sheet_1.DOCX]

| **A**. | 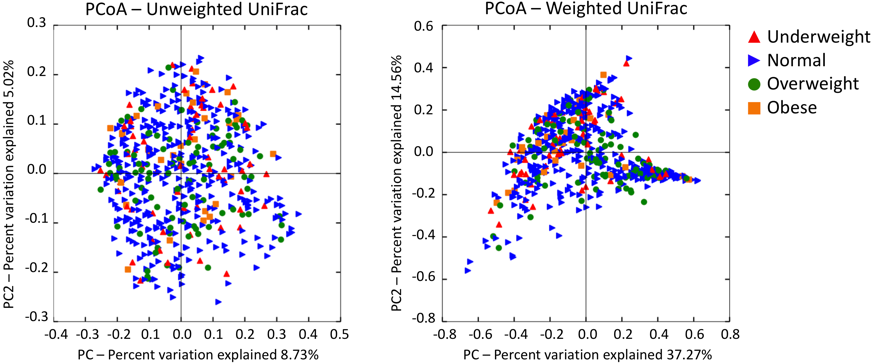 |
| --- | --- |
| **B.** | 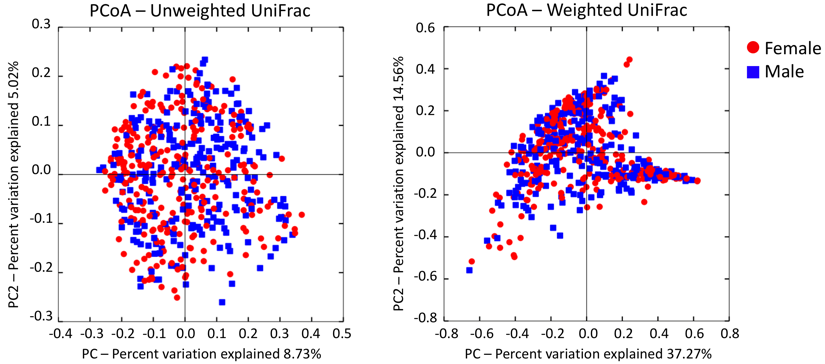 |
| **C.**  **Male** | 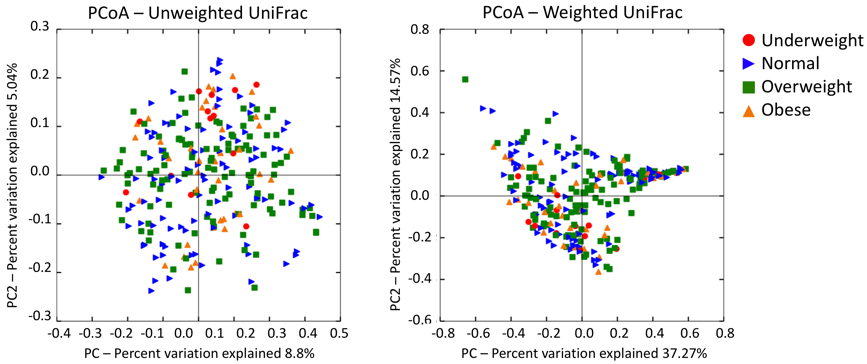 |
| **Female** | 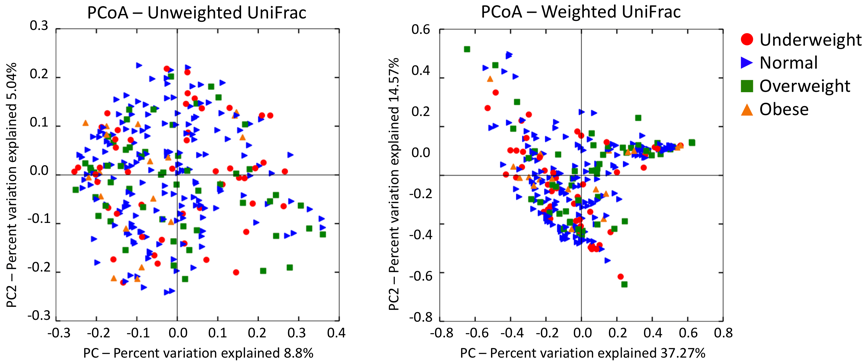 |

**Figure S1**. **Comparisons of** **beta-diversity of gut microbiomes in fecal samples****.** Principal coordinate analysis (PCoA) of bacterial beta-diversity based on unweighted and weighted UniFrac distances of the fecal microbiome shows no clear separation between **(A)** different BMI groups, **(B)** females and males, or **(C)** gender-specific BMI groups**.**

**Figure S2. The genus *Ruminococcus* was significantly higher in Chinese females compared to male subjects.** A generalized linear model was employed to estimate the representation of different bacterial types. Data are shown with Benjamini-Hochberg adjusted p-values. *, P < 0.05; **, P < 0.01; ***, P < 0.001.

| **Overall** | 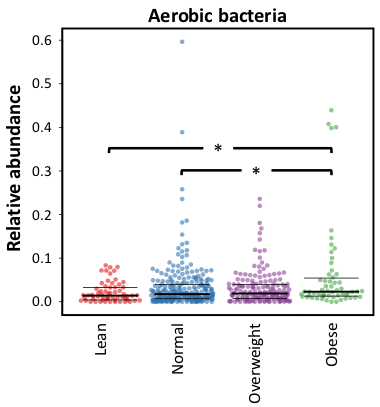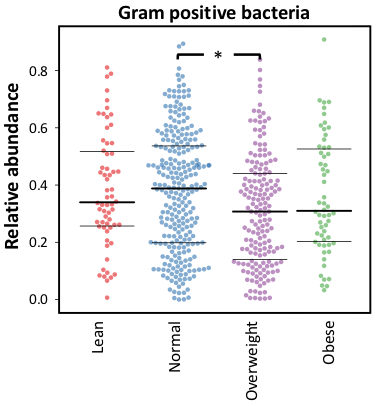 |
| --- | --- |
| **Female** | 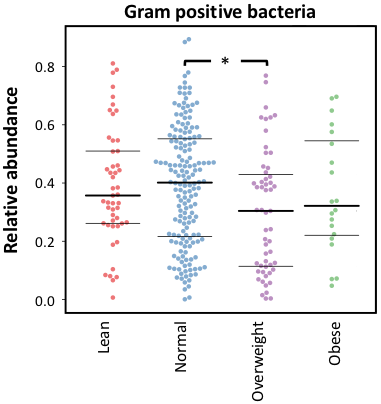 |
| **Male** | 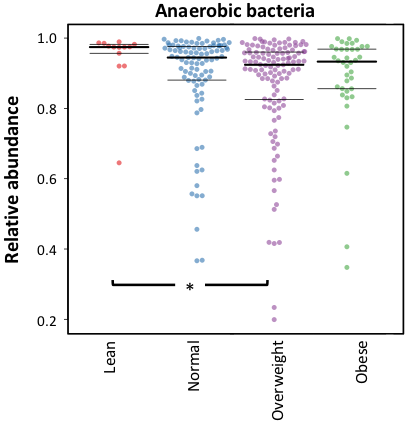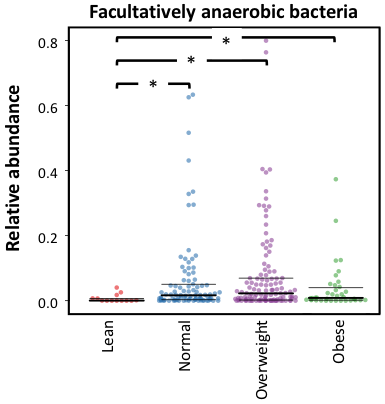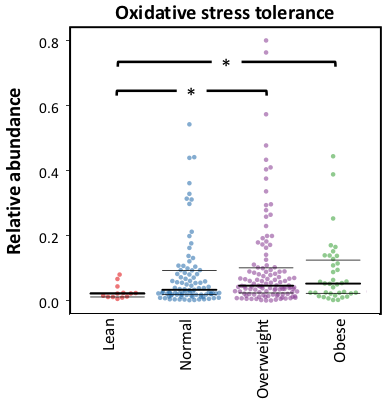 |

**Figure S3. BugBase predicts differences in BMI-associated gut microbiome phenotypes**. Relative abundances for various phenotypes were compared using Mann-Whitney-Wilcoxon tests with false discovery rate correction. *, p < 0.05.


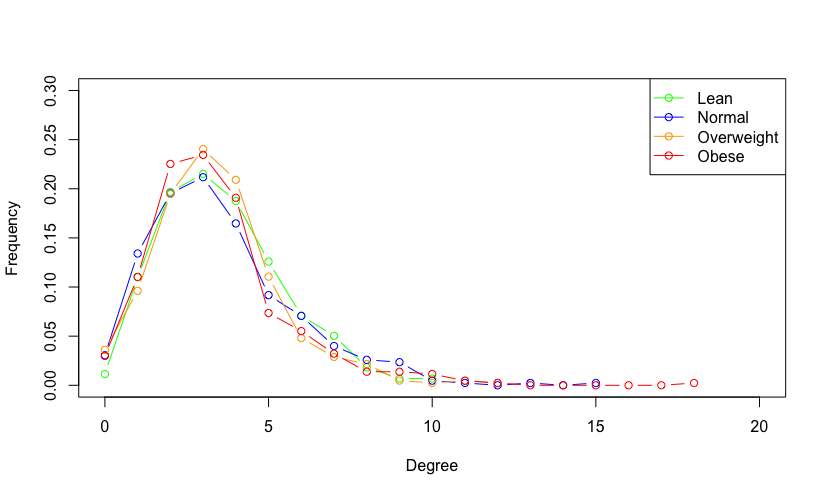


**Figure S4**. **Degree distributions of inferred networks**. The degree of a node is measured by the number of connections (i.e., edges) it has to other nodes.


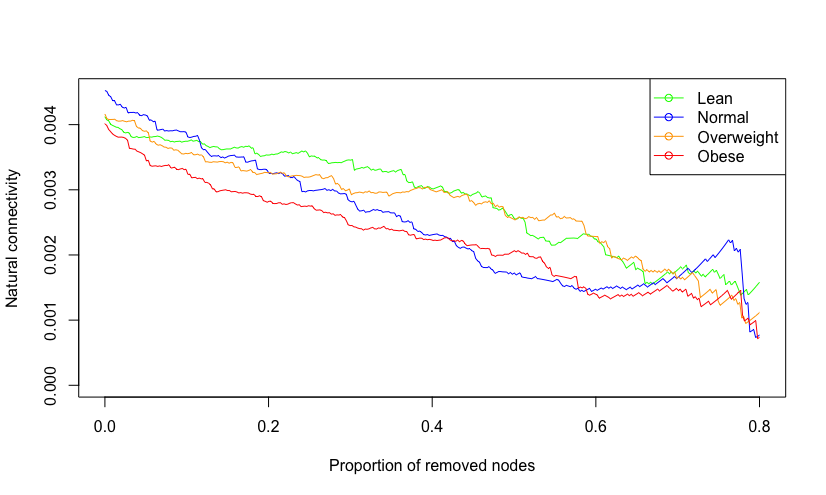


**Figure S5**. **Stability of the inferred network**. Natural connectivity, defined by the average eigenvalue of the graph adjacency matrix as the graph shrinks, was used to assess the robustness of microbial ecological correlation networks to sequential node removals. The node removal method pre-specifies node order by hub characteristics including node degree and betweenness centrality.
